# Association of Cord Blood Levels of Lead, Arsenic, and Zinc and Home Environment with Children Neurodevelopment at 36 Months Living in Chitwan Valley, Nepal

**DOI:** 10.1371/journal.pone.0120992

**Published:** 2015-03-24

**Authors:** Rajendra Prasad Parajuli, Masahiro Umezaki, Takeo Fujiwara, Chiho Watanabe

**Affiliations:** 1 Department of Human Ecology, Graduate School of Medicine, University of Tokyo, 7-3-1, Hongo, Bunkyo-ku, Tokyo, 113-0033, Japan; 2 International Institute for Child Rights and Development (IICRD), PO Box 8646 Victoria Main, Victoria, British Columbia, V8W 3S2, Canada; 3 Basu Laboratory, CINE Building, Macdonald Campus, Faculty of Agricultural and Environmental Sciences, McGill University, Montreal, Quebec, Canada, H9X 3V9; 4 Department of Social Medicine, National Research Institute for Child Health and Development, 2-10-1, Okura, Setagaya-ku, 157-8535 Tokyo, Japan; University of Cincinnati, UNITED STATES

## Abstract

**Background:**

Inconsistent results continue to be reported from studies linking low-level prenatal lead exposure and child development. Because of limited earlier epidemiological studies with birth cohort follow up design, it still remains inconclusive that either the associations of cord blood level of toxic, and essential elements, and postnatal raising environment on neurodevelopment of children remains constant throughout childhood or change over time.

**Aims:**

This study aims to investigate the influence of *in utero* toxic [lead (Pb) and arsenic (As)] and essential elements [zinc (Zn)] levels on neurodevelopment of 36 months children in Chitwan valley, Nepal taking the postnatal environment into account.

**Study Designs and Subjects:**

In this birth cohort study, participants (N=100 mother-infants’ pairs) were recruited in Chitwan district, Nepal. We measured Pb, As and Zn concentrations in cord blood. Postnatal raising environment (i.e., Home score or home environment hereafter) was evaluated using Home Observation for Measurement of Environment (HOME) scale. Neurodevelopment of children at 36 months of age (n=70) were assessed using Bayley Scale of Infant Development, Second Edition (BSID II). Multivariate regression was performed (n=70) to see the association of *in utero* toxic and essential elements level and home environment with neurodevelopment score adjusted for covariates.

**Results:**

Cord blood levels of Pb, As and Zn were not associated with any BSID II cluster scores of 36 months children. The children with relatively superior HOME score and concurrent nutritional status (weight at 36 months) showed better cognitive development (i.e., MDI scores) and motor functions than their counterparts, respectively.

**Conclusion:**

In this general population in Nepal, prenatal Pb, As and Zn levels are not important determinants of the neurodevelopment of 36- month-old children while a consistent beneficial effect of a stimulating home environment on neurodevelopmental indicators is continued.

## Introduction

The detrimental effects of *in utero* toxic elements exposure with later neurodevelopment have been well established at higher exposure levels [1–3]. However, results of epidemiological studies on the association of *in utero* toxic elements exposure with later neurodevelopment at low levels still remain inconclusive [4–6]. For example, in a cohort in Krakow, Poland, cord blood Pb level (mean cord blood Pb = 12.1 μg/L) was inversely associated with the mental development index (MDI) score of the Bayley scale evaluated at 36 months among only boys [6]. In contrast, a cohort study in Boston, USA [7] could not detect such association between cord blood lead and cognitive development at 36 months with higher exposure levels (mean cord blood Pb = 65 μg/L) than the Krakow cohort. Hence, further investigation about the shape of the dose-effect relationship between lead exposure and neurodevelopment at quite low levels will be helpful to understand the controversies that continue to exist.

On the other hand, it remains inconclusive in lower exposure levels whether the associations between cord blood levels of toxic elements or neurodevelopment of children remains persistent throughout childhood or changes over time. For example, in the Krakow cohort [6], cord blood Pb was not associated with BSID II scores at 12 months. However, the association developed to borderline significance at 24 months. By 36 months, the association became significant among boys. In a New Mexico Cohort [8], cord blood Pb (mean cord blood Pb = 67 μg/L) was associated with the MDI score of the BSID II at 24 months. Though, the association became weak when children grow >3 years. In contrast, results from earlier cohort studies indicated that if the prenatal exposure level is high, an inverse association between *in utero* exposure of Pb and neurodevelopmental indicators continues to exist from birth to 3 years consistently [1–3]. We have recently reported the inverse association of *in utero* toxic elements exposure (e.g., Pb mean 20.6 μg/L and As 1.33 μg/L) with neurodevelopment of infants [9] but could not detect such an association at 6 [10] and 24 months [11]. However, it is not clear whether these associations remain constant throughout childhood or change (i.e., re-emerge as delayed onset) by the time of 36 months.

A growing number of epidemiological studies have been conducted to investigate the association between As exposure and neurodevelopment indicators among children [12–17]. Lack of association between maternal As level during pregnancy (i.e., urinary As level during the first, second, and third trimesters as a proxy of prenatal exposure) and neurodevelopmental indicators (BSID II) at 7, 18 and 60 (among boys only) months was reported in Bangladesh [18–20]. However, no study has investigated the association between cord blood As level, which is considered a better bio-indicator of prenatal exposure [9], and the neurodevelopment of 36-month-old children, which is an important developmental milestone including mental and psychomotor development [21].

Regarding the association between *in utero* levels of Zn and neurodevelopment, randomized control trials showed that zinc supplementation to pregnant mothers and infants improves fine and gross motor skills of children in China [22], while some other studies could not detect such effects [23–25]. Inconsistencies among the studies may be attributable to the different levels of Zn, different settings and different populations.

In the present study, we targeted the Chitwan Valley in lowland (Terai) Nepal, because we found inverse associations between cord blood levels of As and Pb, and neurodevelopmental indicators (i.e., motor and state regulation cluster score, respectively) measured by the Brazelton Neonatal Behavioral Assessment Scale, third edition (NBAS III) at birth [9]. However such an association was not evident at 6 [10] and 24 months [11] evaluated by the BSID II. In the current study, we aimed to investigate the influence of *in utero* toxic elements exposure of Pb and As, and the essential element, Zn, considering the effect of home environment on neurodevelopment scores of 36 months old children in Chitwan district, Nepal. Further, this study looked at the time lag effect; i.e., whether the associations of cord blood level of toxic, and essential elements, and postnatal raising environment on neurodevelopment of children remain constant throughout childhood or can change over time.

## Methods

### Study sample

The eligibility criteria to participate in the present study were: living in the study area (i.e., Chitwan) for at least 2 years, at term pregnancy when the mothers visited the hospital (more than 37 weeks of gestation), aged 18 to 40 years, vaginal delivery, singleton, and no report of diabetes, hypertension, and preeclampsia. Two hundred pregnant mothers were approached from September 2008 to October 2008 in the Bharatpur General Hospital of Chitwan district. Among them, 119 were eligible. Mothers were informed of the background and objectives of the study, what they will experience during the study process, benefits to the participants, and potential risks (although not expected). One hundred women signed a letter of informed consent, i.e., participated (participation rate, 84%). The study protocol was approved by the ethics committees of the Graduate School of Medicine, the University of Tokyo (approval no #2244) and of the Bharatpur General Hospital, Chitwan, Nepal.

### Measurements of cord blood elements levels

Cord blood was collected from the placenta by midwives following the common aseptic procedure. Cord blood (10 mL) was collected into a trace metal-free cryovial that contained ethylenediaminetetraacetic acid (EDTA) as an anticoagulant. Cord blood samples were stored in a freezer (-20°C) for less than 1 month, transported to the laboratory in Tokyo (kept frozen with dry ice), and stored in a deep freezer (-78°C) until analysis.

The levels of toxic elements (Pb, and As) and the essential element zinc were measured in cord blood samples in the department of Human Ecology, University of Tokyo. Homogenized whole cord blood (500 μL) was digested with 0.5 mL nitric acid (ultra-pure grade, Wako) in a gas-tight steel container (Uni-seal, San-ai Co. Ltd, Aichi, Japan) at 140°C for 3 h, and the concentrations of the elements were determined with an inductively coupled plasma mass spectrometer (ICP-MS; Agilent 7500 ce, Agilent Technology Tokyo, Japan). Methods and research findings from this cohort have been published previously [9,10,26,27]. The certified reference material (CRM), “Seronorm” trace elements whole blood level-1, lot MR 4206 (Sero AS, Billingstad, Norway), was used. The observed values for each element were within the certified range. Randomly selected cord blood samples (20%) were analyzed twice for all elements. For all the elements measured, there was no statistical difference between the two measurements, and the correlations between them ranged from 0.90 to 0.95, depending on the element.

Of the 100 cord blood samples, 94 samples were used for measurement of toxic elements and the essential element zinc. It was not possible to identify 6 samples due to lack of ID information on the tube tag. In addition, due to limited sample volume, 15 cord blood Pb levels could not be re-measured; thus, 79 cord blood Pb data were used. Out of 94 samples, all cord blood levels for Pb, As and Zn were measured above the detection limit (i.e., 0.026, 0.006 and 2.46 μg/L, respectively).

### Anthropometry of mothers and infants at birth 6, 24 and 36 months

The height and weight of mothers were recorded just before delivery. Body weight was recorded to the nearest 0.1 kg using a portable digital scale (Model BF-046 WH; Tanita, Tokyo Japan). Height was measured to the nearest 0.1 cm. Body mass index (BMI) was calculated by dividing weight (kg) by height squared (m^2^). Birth weight of newborns was obtained from hospital records. Infant’s height and weight were also measured at 6, 24, and 36 months using the same devices.

### Interview at the day of delivery

The following information was collected by interview to mothers after delivery: mother’s age, parity, gender of baby, gestational age, time and date of delivery, educational level, annual income of family, smoking during pregnancy and drinking alcohol during pregnancy.

### Postnatal home environment

The author (RPP) visited the house of each mother/infant pair after approximately 36 months (36.9 ± 0.4 months after their baby was born) after delivery and evaluated the postnatal home environment by the HOME scale [28]. The scale is the total evaluation (i.e., by both observation and interview) of the parent’s response to their child’s behavior, acceptance of child, organization of environment, learning material, parental involvement with the child, and opportunities of variety for babies by 45 items. Thus, possible scores range from 0 to 45, with scores < 25 indicating a less stimulating home environment [29]. Of the 100 mothers enrolled in the cohort, home environment of 74 houses was evaluated by a single evaluator (by one of the author: RPP).

### Neurodevelopmental indicators of 36 month old children

The second edition of the Bayley scale of infant development (BSID II) [30] was used to assess neurodevelopmental status of 36 month old children. The BSID II scale has been frequently used in the field of neurotoxicology [6,29,31–37]. The BSID II provides two neurodevelopmental indicators: the MDI and the Psychomotor Development Index (PDI). MDI reflects the infant’s level of cognitive function, language, and personal or social development. PDI reflects the gross and fine motor functions.

The BSID II test was administered to the children within 8 weeks of the target age group (i.e., 36 months ± 2 month; as accepted by BSID II manual) and the age of children in days were recorded. The BSID II assessment of all children was conducted in their own house by the author (RPP) blinded to the exposure status. Assessment of children in their own house judged to be convenient to the caregiver as well as for the cohort children (no shyness because of familiar environment). Uniform assessment conditions [i.e., early information about assessment, limited presence of family members or siblings, friendly approach of tester to children, moderate light at the place of assessment, with fresh mood of children (i.e., no hunger or sleepiness)] was maintained.

### Statistical Analysis

Firstly, distribution of all the variables was examined for normality. Cord blood levels of toxic and essential elements and annual family income were log-transformed.

Bivariate (Model 1) and multivariate (Model 2) analyses were conducted to examine the (unadjusted and mutually or fully adjusted association, respectively) associations between neurodevelopment indicators and toxic (Pb and As) and the essential element zinc and the HOME scale score [18,25,29,33,38,39] and covariates [mother’s age [40], parity [35–37], mothers education level [6,34–37,41–44], family income [45], mother’s BMI just before birth [18], weight of children at birth and at 36 months after birth [18,25,29,42], gestational age [18,46], and children’ age at the time of BSID II assessment [29]] that are known to correlate with the neurodevelopmental indicators. In Model 3, four covariates (i.e., parity, family income, mother’s BMI just before delivery, and weight of the infant at birth) were dropped from the fully adjusted model (Model 2) to see the “minimum” adjusted effect of explanatory variables and covariates, on response variables.

The distribution of the covariate, age at BSID II assessment in days, was also normal. Smoking during pregnancy and drinking alcohol during pregnancy were not analyzed because only 5 smoked and 4 drank out of 100 mothers.

The *p*-values less than 0.05 were considered as statistically significant. Statistical analyses were performed using SPSS version 11.5 (SPSS Inc., Tokyo, Japan).

## Results

### Characteristics of mothers and children

Table 1 summarizes the characteristics of mothers and child pairs at birth, at 6, 24 and 36 months after birth. Maternal, household and newborns characteristics from this cohort have been published previously [26,27]. Indicators for nutritional status of newborns (i.e., birth weight, height) did not differ by sex. In contrast, weight of children at 36 months after birth differed by sex (*p*<0.03, i.e., boys were in average 670 grams heavier than girls, data not shown). The mean body weight of the present study children at 36 months after birth was 12.7 kg (ranged 9.6 kg to 16.5 kg). Body height became similar between girls and boys children at 36 months after birth.

**Table 1:** Characteristics of the mothers and infants who participated in the study.

Characteristics		Mean or N (SD or %)	Range
**Mothers characteristics at birth (n = 100)**			
	Age (years)	22.9 (3.7)	18 to 37
	Primipara	66 (66.0)	
	Education level (years)	9.2 (3.8)	0 to 17
	BMI (kg/m2)	23.2 (2.9)	16.8 to 32.7
**Newborn babies characteristics (n = 100)**			
	Gestational age (weeks)	38.9 (1.4)	37.0 to 43.0
	Sex of baby (male)	47 (47.0)	
	Birth weight (g)	3029 (438)	2200 to 4000
	*Cord blood elements (Median)*		
	Pb (μg/L) (n = 79)	20.6	6.83 to 220.8
	As (μg/L) (n = 94)	1.33	0.51 to 9.58
	Zn (μg/L) (n = 94)	2112	1299 to 6430
**Household Characteristics**			
	Annual family income in US Dollar (USD)	2529 (2882)	150 to 17250
	Total HOME Scale score at 36 months (n = 74)	37.9 (7.3)	19 to 51
**Infants characteristics at 36 months after birth (n = 70)**			
	Body weight (kg)	12.7 (1.5)	9.6 to 16.5
	Height (cm)	91.5 (4.0)	81 to 100
	**Scores on BSID II clusters**		
	Mental Development Index (MDI) score	95.1 (10.0)	61 to 119
	Psychomotor Development Index (PDI) score	114.6 (9.1)	81 to 133
	Age at BSID II assessment in 36 months (in months)	36.9 (0.40)	36.1 to 37.6

### Neurodevelopmental indicators of 36 month old children

All the children were evaluated by the BSID II scale at 36 months after birth (mean assessment age = 36.9 months). These index scores of the cohort children were mostly normally distributed. The MDI score in the BSID II observed in this cohort [i.e., 95.1] was lower than the scores reported by Krakow cohort [6] in Poland [i.e., 103.1] but similar to the MDI score reported in the Mexico cohort [93.2] [47] and New York City cohort [90.0] [48]. The PDI score in this cohort [i.e., 114.6] was higher than previously reported scores [i.e., 95.8] in the Mexico Cohort [47] and New York City cohort [i.e., 100.5] [48]. All the cluster scores did not differ by gender (*p*>0.05, data not shown). According to criteria provided by the BSID II manual [30], MDI score for 1 child (1.4%) fell into the “significantly delayed development” range (i.e. 69 and below), while 8 children (11.4%) fell into the range of “mildly delayed development” (i.e., 70–84). Scores of PDI for 1 child (1.4%) was categorized as “significantly delayed development (i.e. 69 and below)” (data not shown).

### Associations among cord blood level of toxic elements and the essential element zinc and BSID II index score

Table 2 summarizes the association among cord blood levels of toxic elements or the essential element zinc, and BSID II index scores. In the bivariate regression model, cord blood toxic elements levels (Pb and As) or the essential element zinc were not associated with any BSID II cluster scores.

**Table 2:** Association of demographic and *in utero* chemical and home environmental variables with MDI and PDI scores of BSID II at 36 months from birth (n = 70).

Response Variables	Mental Development Index Score			Psychomotor Development Index Score		
Explanatory variables	Model 1^a^	Model 2^b^	Model 3^c^	Model 1^a^	Model 2^b^	Model 3^c^
***Cord blood level of*** *(log μg/L)*	Estimated change (95% confidence interval) in the value of the response variable for a one unit increase of the explanatory variable					
Lead (Pb)	5.21 (-2.80 to 13.22)	4.05 (-3.21 to 11.31)	4.46 (-2.46 to 11.37)	-1.77 (-9.16 to 5.62)	-2.56 (-9.71 to 4.59)	-1.68 (-8.42 to 5.06)
Arsenic (As)	1.63 (-11.09 to 14.34)	4.89 (-7.21 to 17.00)	2.55 (-9.07 to 14.17)	7.27 (-4.21 to 18.75)	7.34 (-4.58 to 19.26)	6.25 (-5.08 to 17.58)
Zinc (Zn)	12.14 (-12.64 to 36.91)	7.00 (-16.42 to 30.42)	2.29 (-19.81 to 24.40)	5.35 (-17.39 to 28.08)	-1.82 (-24.90 to 21.25)	-2.43 (-24.00 to 19.12)
HOME Score at 36 months	**0.53 (0.23 to 0.84)**	**0.66 (0.27 to 1.05)**	**0.69 (0.31 to 1.07)**	0.27 (-0.03 to 0.57)	0.22 (-0.16 to 0.60)	0.27 (-0.10 to 0.64)
**Covariates**						
Mother’s age (years)	-0.14 (-0.77 to 0.49)	-0.02 (-0.85 to 0.82)	-0.26 (-0.85 to 0.33) (	-0.19 (-0.77 to 0.39)	0.07 (-0.75 to 0.89)	0.19 (-0.77 to 0.39)
Parity	-1.80 (-4.69 to 1.08)	-0.76 (-4.95 to 3.44)	-	-1.51 (-4.15 to 1.13)	-1.36 (-5.49 to 2.78)	-
Mothers education level (years)	0.60 (-0.05 to 1.24)	-0.10 (-0.91 to 0.71)	-0.14 (-0.85 to 0.58)	0.41 (-0.19 to 1.00)	0.11 (-0.69 to 0.90)	0.08 (-0.62 to 0.78)
Log annual family income (USD)	1.56 (-4.77 to 7.89)	-4.68 (-11.37 to 2.01)	-	2.98 (-2.76 to 8.73)	-3.32 (-9.91 to 3.27)	-
Mothers BMI (kg/m2) just before delivery	0.50 (-0.27 to 1.28)	0.39 (-0.37 to 1.16)	-	0.37 (-0.34 to 1.07)	0.36 (-0.39 to 1.11)	-
Birth weight (kg)	-0.63 (-5.85 to 4.59)	-1.92 (-7.04 to 3.19)	-	2.51 (-2.22 to 7.24)	1.37 (-3.66 to 6.41)	-
Weight of infants at 36 months (kg)	1.09 (-0.53 to 2.71)	1.26 (-0.32 to 2.85)	0.88 (-0.60 to 2.36)	**2.13 (0.73 to 3.53)**	**2.15 (0.60 to 3.71)**	**2.10 (0.66 to 3.55)**
Gestational age (week)	1.52 (-0.23 to 3.27)	0.99 (-0.62 to 2.59)	0.87 (-0.68 to 2.42)	1.40 (-0.20 to 3.00)	0.95 (-0.63 to 2.53)	0.86 (-0.65 to 2.37)
Age at BSID II assessment (days)	**-7.67 (-13.65 to 1.70)**	**-12.06 (-18.06 to -6.06)**	**-11.40 (-17.09 to -5.71)**	-5.24 (-10.81 to 0.33)	**-9.28 (-15.19 to -3.37)**	**-8.54 (-14.09 to -2.99)**
R^2^		0.423	0.389		0.329	0.302

Bivariate and multivariate associations between *in utero* chemical and home environmental variables and covariates with BSID II clusters were shown in the table.

Model 1^a^: Bivariate regression analysis was conducted to see the unadjusted association between all the “explanatory variables (*in utero* Pb, As, and Zn level and HOME Score)” and “covariates (i.e., mother’s age, parity, mother’s education level, family income, mother’s BMI just before delivery, weight of the infant at birth and 36 months after birth, gestational age and infant’s age at the time of BSID II assessment)”.

Model 2^b^: Multivariate regression model was conducted to see the mutually adjusted effect of explanatory variables, and covariates, on response variables. Out of 100 cohort mother infant pairs recruited, although cord blood Pb level was available for 79 participants, and HOME score was available for 74 participants, only 70 participants (n = 70) who had complete sets of data available for analysis were considered in the model.

Model 3^c^: In this model, “parity, family income, mother’s BMI just before delivery, weight of the infant at birth” were dropped from fully adjusted model (Model 2) to see the “minimum” adjusted effect of explanatory variables, and covariates, on response variables.

**Fig 1:**
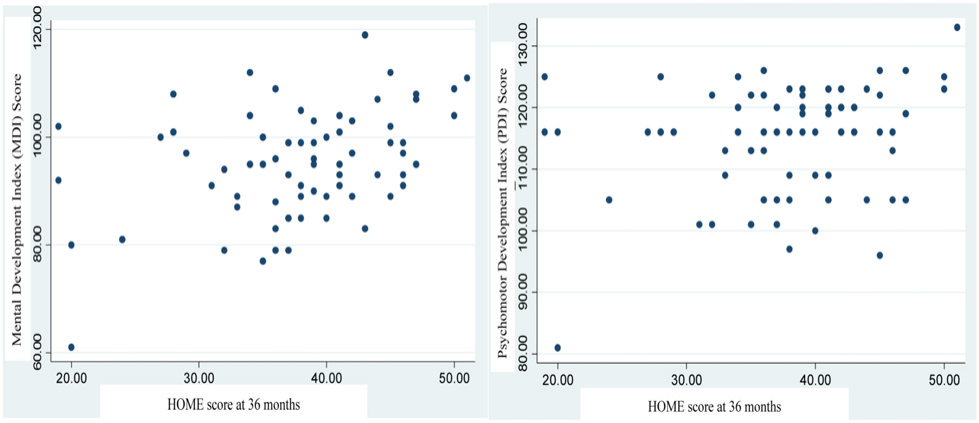
BSID II cluster scores (MDI and PDI) by HOME score are shown. BSID II: Bayley Scale of Infant Development, Second Edition. HOME Score: Home Observation for Measurement of Environment scale score. MDI: Mental Development Index, PDI: Psychomotor Development Index.

Total HOME scale scores positively associated with MDI scores (Fig. 1). Weight of children at 36 months was positively associated with PDI scores, while age at BSID II assessment was inversely associated with MDI and PDI scores.

In the multivariate regression model (Table 2), cord blood toxic elements (Pb and As) levels or the essential element zinc were not associated with any BSID II cluster scores. Home environment remained positively associated with MDI score. Concurrent nutritional status of children (i.e., weight of children at the 3 year visit) also remained significantly associated with fine and gross motor functions of children (i.e., PDI score). In addition, the minimally adjusted model (Model 3) and fully adjusted model (Model 2) showed similar results in terms of direction and significance despite some differences in coefficients and R square values. There were no significant associations concerning interactions between toxicants (for example, Log Pb and Log As) as well as interactions between HOME and the neurotoxins or the essential elements (for example, HOME score and Log Zinc level).

## Discussion

In this cohort, cord blood levels of Pb, As and Zn were not associated with any BSID II cluster scores of 36 months old children in multivariate models. The children with relatively higher HOME scores and better concurrent nutritional status showed better cognitive development (i.e., MDI scores) and motor functions (i.e., PDI scores) than their counterparts, respectively.

### Associations among cord blood levels of toxic elements and BSID II cluster score

Cord blood levels of toxic elements (i.e. Pb and As) were not associated with any BSID II cluster scores evaluated at 36 months. The cord blood levels of Pb and As showed a significant inverse association with the motor and regulation of state cluster score (NBAS III) of the cohort infants evaluated at birth [9] but such an association did not continue to exist at 6 [10] or 24 months [11]. In contrast, results from earlier cohort studies indicated a consistent inverse association between *in utero* exposure of Pb and neurodevelopmental indicators continued to exist from birth to 3 years in relatively higher (>63 μg/L) Pb exposure levels [1–3]. Hence, the harm of *in utero* exposure to Pb (20.6 μg/L) on the neurodevelopment of infants might be too small to persist until age 6 or 24 or 36 months. However, even in low exposure levels (12.3 μg/L), an inverse association between prenatal Pb level and BSID II scores at 36 months has been reported in the Krakow cohort study (among boys only) in Poland [6,36]. The possibility of lack of power [as inferred in the Mexico cohort (n = 83) [8,49]] because of a small sample size in our cohort (n = 70), compared to the bigger sample size (n = 457) in Krakow cohort, cannot be ignored for the negative finding.

The cord blood levels of Pb and As, showed a significant inverse association with the motor and regulation of state cluster score (NBAS III) of the cohort infants evaluated at birth [9]. Diminution of effect over time during postnatal period as inferred by Claus Henn et al. [50] by shedding (i.e., excretion) most of the As burden from the child body via urine [51] may be one possible pathway. Neuroplasticity of the immature brain might be another possible reason behind the diminution of effect over time [52]. A similar mechanism can be speculated for Pb as well.

### Associations among cord blood levels of the essential element zinc and BSID II cluster score

Cord blood level of essential elements (i.e. Zn) was not associated with any BSID II cluster scores. Similar to earlier studies [23–25], children with higher *in utero* Zn levels did not show better neurodevelopment scores in this cohort. This may be because of good nutritional status of cohort babies as evident by their birth weight or the BSID scale is not able to recognize the subtle effect of Zn in its index score. Further, concurrent nutritional status (weight of children at 36 months) also contributed significantly in motor functions of 36 months old children.

### Associations between the HOME scale score and BSID II cluster score

The HOME scale score was positively associated with gross and fine motor development (MDI) at 36 months after birth, which is in line with the observation in Mexico [29], but not in the United States cohort [32]. The positive association between the HOME scale score and neurodevelopmental indicators were also consistently reported among elder (18 to 24 months) Latino children in the United States [53] and children of native German families [54]. Interestingly, at 6 months the HOME scale score was positively associated with motor development (i.e., PDI score) while at 36 months it was associated with mental development (i.e., MDI score). This may be because of an increased capacity of children to interact with their surrounding environment with older age. At 6 months, home environment in terms of “organization of environment” and “learning material for babies” in the HOME cluster contributed to lower PDI scores [10]. However, at 36 months of age, we can speculate a better (than that of early age) children’s response towards the parental dealing (i.e., “response to child’s behavior,” “acceptance of child,” and “involvement with the child” aspects of the HOME scale) with children. At an older age, children can interact with ‘mentioned stimuli in terms of parental involvement’ for improved mental development (i.e., improved MDI). In addition, because of cultural assimilation among different cultural and religious groups such as the Hindus and Buddhists, Nepalese children are celebrating many festivals together, which involve different family visits. Such frequent family visits with babies might provide the babies with better “opportunities of variety” to learn as described in the HOME cluster scores.

### Limitations

Several limitations should be considered before conclusion. First, the small sample size and hospital based sampling may limit the generalizability of the finding. In addition, the small sample size limits us in estimating an interaction effect between toxicants with confidence. Second, several potential confounders were not measured. They include prenatal exposure to mercury and pesticides [29,33]. They should be included in the future study. Third, applicability of the HOME scale in the Nepali context can be another problem. A few items (i.e., 5 out of 45 items) in the HOME scale e.g., “availability of specific toys” might cause underscoring for the HOME score since such toys are not common in Nepal. Yet, the HOME scale has been frequently used in developing countries [55–57]. Despite the ease in collection and frequent use, because of increased body fluid near the delivery, some concern has been raised for cord blood as an index for *in utero* exposure. However, the effect of increased body fluid near the delivery on metal concentration has not yet been precisely evaluated.

Nonetheless, this study has a number of strengths. This cohort is the first longitudinal birth cohort from Nepal for which considerable information was collected about environmental exposures and potential confounders. Our population had a wide range of and relatively higher average As and Pb levels than the exposure levels reported from population with background exposure levels in developed countries. BSID II was evaluated by single tester at the participant’s home, which may avoid the inter-tester bias together with possible underperformance by children in a new assessment environment. Future epidemiological research should be conducted to evaluate the effect of *in utero* and postnatal exposure of toxic and essential elements on neurodevelopment in other populations from developing countries with different exposure/ deficiency levels.

## Conclusions

In a birth cohort study in the Terai region, lowland Nepal, exposure of Pb, As and deficiency of Zn were not high enough to cause detrimental effect on the neurodevelopment of 36 month old children in Nepal. While a consistent beneficial effect of a stimulating home environment on neurodevelopmental indicators is continued.
